# The role of silver ions in the regulation of the senescence process in Triticum aestivum

**DOI:** 10.3906/biy-1802-95

**Published:** 2018-12-10

**Authors:** Mert ÖKTEM, Yüksel KELEŞ

**Affiliations:** 1 Department of Biology Education, Faculty of Education, Mersin University , Mersin , Turkey; 2 Department of Biotechnology, Institute of Sciences, Mersin University , Mersin , Turkey

**Keywords:** Dark, indole-3 acetic acid, silver nitrate, antioxidants, pigments

## Abstract

The control of senescence has economic importance due to its effects on parameters such as herbal product quality and shelf life. This study is on the control of induced senescence in Triticum aestivum L. 'Gün-91' plants with silver nitrate (AgNO3) treatments. It was observed that some changes that occurred with dark and indole-1-acetic acid (IAA) treatments could be reduced with AgNO3 treatments. After dark-induced senescence, it was observed in plants that seedling length, relative water content (RWC), chlorophyll, β-carotene, xanthophylls, total antioxidant capacity, soluble phenol, total soluble protein, catalase (CAT), total superoxide dismutase (SOD), copper-zinc superoxide dismutase (Cu/Zn-SOD) activities, and expression of genes encoding these enzymes declined. After IAA treatments, seedling length, RWC, chlorophyll, β-carotene, xanthophylls, total antioxidant capacity, soluble phenolics, and soluble protein levels declined, whereas activities of CAT, total SOD, and Cu/Zn-SOD enzymes and expression of Cu/Zn-SOD and CAT genes increased. AgNO3 (200 mg L-1 ) applied by spraying onto leaves led to an increase in seedling length, RWC, chlorophyll, β-carotene, xanthophylls, total antioxidant capacity, soluble phenolics, soluble protein levels, and expression of Cu/Zn-SOD, CAT genes, CAT, SOD, and Cu/Zn-SOD enzyme activities compared to controls. Findings obtained from this study showed that the senescence process was related to changes in the levels of antioxidant compounds and enzymes. It was defined that the role of silver ions in slowing senescence was related to antioxidant defense capacity.

## 1. Introduction

Senescence effects many different agriculturally important properties such as seed count, yield and quality, fruit maturation, seed germination time, and postharvest shelf life (Liebsch and Keech, 2016) . Early senescence leads to a 50% decrease in yield of grains (Gan, 2007) . Senescence is defined as the transport of nutrients and minerals in aging tissues to developing tissues of the plant. In this process, expression of genes associated with photosynthesis decreases, whereas expression of genes associated with destruction and transportation of macromolecules increases (Buchanan-Wollaston et al., 2003) . In plants, senescence is characterized by visibly yellowed leaves. Yellowing of leaves progresses with several biochemical processes such as loss of chlorophyll content, degradation of proteins and RNA, and decreased photosynthetic activity (Lim et al., 2007; Keles, 2009) .

Reactive oxygen species (ROS) rapidly increase with the onset of leaf senescence. The increase in superoxide anion (O2-.), hydrogen peroxide (H_2_O_2_), hydroxyl radical (OH), singlet oxygen (1O2), and other harmful free radicals causes membrane lipid peroxidation, cellular damage, and programmed cell death (Prochazkova and Wilhelmova, 2007; Breusegem and Dat, 2006) . High grain yield is obtained from wheat types that remain green throughout grain filling (Zhang et al., 2006) , while low grain yield is obtained from early senescing wheat types due to reduction in photosynthesis (Hongwei et al., 2014). Decreased protein synthesis and antioxidant enzyme activity in the senescence process increase ROS damage. Treatment with 2,4-D, an auxin derivative, effects antioxidant enzymes such as POX and SOD and AA-GSH cycle enzymes such as MDHAR, DHAR, and GR and increases their activity (Manoharan et al., 2005) .

High concentrations of 2,4-D in plants induce senescence. Auxin treatment stimulates 1-aminocyclopropane-1-carboxylic acid synthase (ACS) activity in plants and subsequent increases in ACS and ethylene levels lead to growth inhibition and induction of senescence (Karuppanapandian et al., 2011) .

The inhibiting effect of Ag + ions on ethylene has been reported by many researchers (Wagstaf et al., 2005; Nejatzadeh-Barandozi et al., 2014) . AgNO3 inhibits ethylene biosynthesis in vitro in cotton and causes regeneration of hypocotyl segments with shoots (Ouma et al., 2004) . In calamondin fruits, ethylene leads to an increase in chlorophyllase enzyme activity and chloroplast membranes are destroyed; however, subsequent 100 mg L–1 AgNO3 treatment prevents chlorophyll destruction by reducing ethylene synthesis (Purvis, 1980) . When applied in the form of AgNO and silver nanoparticles (AgNPs), 3 Ag+ ions inhibit senescence in beans. 2,4-D-induced leaf senescence is more efficiently inhibited by supplementation with AgNPs than AgNO3 (Karuppanapandian et al., 2011) . Supplementation with ethylene inhibitors such as AgNO3 (Strader et al., 2009) and silver thiosulfate (Giridhar et al., 2001) increases shoot formation by delaying aging. In wheat, spraying of AgNO3 leads to an increase in grain yield (Labrana and Araus, 1991) . AgNPs and AgNO3 sprayed onto Ocimum basilicum plants were observed to lead to an increase in seed yield (Fatemeh et al., 2014).

In this study, the concentration of antioxidant compounds, activity of antioxidant enzymes, and change in expression of genes encoding these enzymes were investigated in indole-3 acetic acid (IAA)- and dark induced senescence processes in wheat. Changes in antioxidant compounds, antioxidant enzyme activities, and antioxidant gene expressions in the senescence process were investigated with AgNO3 treatment in wheat. Ag+ ions are known to be effective on senescence. However, there is not adequate research on effects of these treatments on the antioxidant defense system. According to the hypothesis constituting the basis of this study, senescence inducers trigger oxidative stress, whereas senescence inhibitors suppress oxidative stress. This may be determined with changes in activities of antioxidant compounds and enzymes and the expression of genes encoding these enzymes. Findings obtained in this study may provide information on the senescence-antioxidant relation and also contribute to the source of knowledge necessary for research on control of the senescence process, agricultural production, and plant biotechnology.

## 2. Materials and methods

### 2.1. Plant materials

In this study, bread wheat (Triticum aestivum L.), which is a species with economic value, was used. Since wheat grain productivity is closely related to monocarpic senescence processes, it is a suitable plant for senescence studies. Gün-91, widely grown in Turkey, is a culture variety that is resistant to cold and drought. Wheat seeds were obtained from the Central Research Institute for Field Crops in Ankara, Turkey. After germinating wheat seeds with soaked filter paper in a petri dish, they were taken to pots containing soil/turf/manure (2/1/1 ratio). Plants were cultivated in a climate chamber under controlled conditions: 16/8 day/night cycle, 26 ± 2 °C day and 18 ± 2 °C night temperatures, 480 µmol m–2 s–1 light intensity, and 65 ± 5% relative humidity. After growing seedlings for 3 weeks, a total of 40 pots each containing 25 seedlings were divided into eight treatment groups of five. These groups were treated with light (control), light + AgNO3 (200 mg L–1), dark, dark + AgNO3, light + IAA (50 and 100 mg L–1), or light + IAA + AgNO3 (Table 1). The IAA and AgNO3 spraying process was repeated consecutively for 14 days with an interval of 1 day. The concentration of AgNO3 suitable for the spray method was determined by literature investigations. After treatment, seedling length and relative water content (RWC) measurements of plants were performed at the end of the 14th day. After freezing plant leaves with liquid nitrogen for later use in analyses, they were stored in freezers at –80 °C.

**Table 1:** Changes in growth, RWC, and pigment content in the dark- and IAA-induced senescence process in wheat. Data represent the means of three replicate ± SD. Statistics: inducers (IAA and dark), silver (AgNO3 treatment), *P ≤ 0.05 and **P ≤ 0.01.

Treatments	Seedling length (cm)	RWC (%)	Total chlorophyll (μg g–1 FW)	β-Carotene (μg g–1 FW)	Total xanthophyll (μg g–1 FW)
Light	35.0 ± 1.0	70.4 ± 1.9	3025 ± 17	110.0 ± 6.0	240.0 ± 9.2
Light + AgNO3	35.3 ± 3.5	65.3 ± 2.5	3869 ± 53	134.7 ± 9.0	260.7 ± 36.0
Dark	29.7 ± 0.6	63.3 ± 3.9	1512 ± 95	80.7 ± 1.2	208.0 ± 2.0
Dark + AgNO3	46.3 ± 2.1	68.7 ± 1.5	2330 ± 96	91.0 ± 22.7	227.7 ± 11.4
IAA(50)	38.0 ± 2.6	64.1 ± 5.1	239 4± 59	86.7 ± 16.2	196.0 ± 35.5
IAA(50) + AgNO3	37.7 ± 1.5	60.9 ± 2.0	2879 ± 65	98.0 ± 11.1	214.7 ± 48.4
IAA(100)	37.7 ± 2.5	68.2 ± 2.3	2126 ± 77	82.0 ± 3.5	182.0 ± 67.7
IAA(100) + AgNO3	39.7 ± 0.6	68.7 ± 11.5	2663 ± 43	95.3 ± 7.0	200.0 ± 81.5
Statistics	Inducer (*)		Inducer (*)	Silver (*)	
	Silver (*)		Silver (*)		

### 2.2. Plant height and relative water content

Five seedlings were randomly picked from each pot and cut from soil level for length measurement. Fresh weight (FW) of the tissue sample was determined. The sample tissue was kept floating in pure water for 2 h and allowed to absorb water until saturation. After drying the surface with filter paper, turgid weight (TW) was measured, and after drying the sample in an oven at 85–90 °C, dry weight (DW) was measured. RWC was calculated using the formula RWC = 100 × [(FW – DW) / (TW – DW)].

### 2.3. Pigment contents

Chlorophyll extraction from fresh leaf material was carried out with 80% acetone (buffered to pH 7.8 with phosphate buffer). Chlorophyll measurements were done with a spectrophotometer. Chlorophyll contents were calculated according to Porra et al. (1989) .

Fresh leaf materials (0.5 g) were ground in a prechilled mortar in 5 mL of acetone containing 200 mg of Na2SO4 and then filtered through glass fiber disks (Whatman GF/A). The volume of the acetone extracts was reduced in a rotary evaporator and then resuspended in 1 mL of chloroform. Fifty microliters of extracts and standards were applied to silica gel TLC plates (20 × 20, 0.25 mm thickness). Hexane, diethyl ether, and acetone were used at 60:30:20, v:v:v, as the mobile phase (Moore, 1974) . Xanthophyll and β-carotene spots were scraped from the TLC plates and centrifuged in 5 mL of acetone for 5 min at 5000 × g. The absorbance of supernatants was determined at a wavelength of 450 nm by a spectrophotometer against β-carotene and lutein standards.

### 2.4. Total antioxidant capacity

Plant samples of 0.5 g were crushed in a mortar with 5 mL of methanol (96%) for determination of  total antioxidative capacity. The extract was centrifuged for 5 min at 5000 × g and the supernatant  was taken. A reactive solution containing 6 M sulfuric acid, 28 mM sodium phosphate, and 4 mM ammonium molybdate was prepared, and then 150 µL of supernatant was mixed with the reactive solution in a test tube so that the final volume would be 3 mL. The tubes was maintained at 95 °C for 90 min and then cooled to room temperature and their absorbances were measured at 695 nm.  Total antioxidative capacity was calculated as the equivalent of ascorbic acid (Prieto et al., 1999) .

### 2.5. Soluble phenolics content

The frozen leaf samples (0.5 g) were rapidly plunged into 20 mL of 80% aqueous ethanol and boiled for 5 min. After filtration through Whatman No. 1 filter paper, ethanol was eliminated from the filtrate by evaporation in vacuum. Total soluble phenolics in the remaining water phase were determined spectrophotometrically with the Folin– Ciocalteu reagent (prepared by 1:1 dilution with distilled water), against the chlorogenic acid standard (Ferraris et al., 1987) .

A sample of 0.5 g of frozen leaf material was shaken in 10 mL of 80% cold acetone for 10 min and centrifuged at 10,000 × g for 10 min. The same process was repeated with precipitate in 5 mL of 80% acetone, and the pellet was suspended in 2 mL of Tris-HCl buffer (0.1 M, pH 8), shaken for 5 min, and centrifuged at 3000 × g for 5 min. The supernatants were collected and stored at –20 °C for soluble protein determination. Soluble protein was determined according to Lowry et al. (1951) .

### 2.6. Enzyme extraction

Frozen leaf material (0.5 g) was homogenized in 6 mL of 0.1 M potassium phosphate extraction buffer (pH 7, containing 100 mg of insoluble PVP and 0.1 mM EDTA) with an Ultra-Turrax. The homogenate was centrifuged for 5 min at 6000 × g and 4 °C. The supernatant was filtered through a Whatman GF/A glass fiber disk with a vacuum filtration system and stored at –70 °C (Schöner and Krause, 1990) .

#### 2.6.1. Catalase activity (CAT, EC 1.11.1.6)

CAT activity was assayed at 20 °C in a reaction volume of 3 mL containing 2.8 mL of 50 mM potassium phosphate buffer (pH 7, not containing EDTA), 120 µL of enzyme extract, and 80 µL of 0.5 M H_2_O_2_. Activity was determined by UV spectrophotometer at 240 nm, measuring the decrease in absorbance for 30 s (Aebi 1983) .

#### 2.6.2. Superoxide dismutase activity ( SOD, EC 1.15.1.1)

SOD activity was determined according to Beyer and Fridovich (1987) . The reaction mixture (3 mL) contained potassium phosphate buffer (pH 8, 0.025% Triton X-100, and 0.1 mM EDTA), enzyme extract, 12 mM L-methionine, 75 µM nitroblue tetrazolium chloride (NBT), and 2 µM riboflavin. The reaction mixture was kept under fluorescent light for 10 min at 25 °C. One SOD unit was described as the amount of enzyme where the NBT reduction ratio was 50%. NBT reduction ratios were measured with a spectrophotometer adjusted to 550 nm.

### 2.7. Gene expression analysis

Total RNA from plant tissues was isolated using TRIzol reagent (Life Technologies, USA) according to the manufacturer’s instruction. RNA quality was tested by agarose gel electrophoresis using standard protocols. Two micrograms of total RNA was used to synthesize cDNA using a First Strand cDNA Synthesis Kit (Roche, Cat. No. 04897030001) following the manufacturer’s protocol. After cDNA synthesis, the amount of cDNA was quantified by NanoDrop ND-1000 spectrophotometer. The cDNA samples were used as templates to quantify target gene expression levels.

The T. aestivum CAT gene (accession number: D86327.1), and the T. aestivum Cu/Zn SOD gene (accession number U69632.1), and the housekeeping gene T. aestivum GAPDH (glyceraldehyde 3-phosphate dehydrogenase) (accession number: AF251217.1) were used for real-time quantitative PCR analysis. The primer pairs for CAT, Cu/Zn SOD, and GAPDH were designed using Primer 3 software (http://bioinfo.ut.ee/primer3-0.4.0/). Genespecific primers were chosen so that the resulting PCR product had approximately the size of 180–230 bp. The primer sequences used were as follows.

GAPDH: (F) 5’-GGAGGAGTCTGAGGGAAACC-3’ (R) 5’-GTGCTGTATCCCCACTCGTT-3’ Cu/Zn SOD: (F) 5’-CTCCTGGACTTCATGGCTTC-3’ (R) 5’-CATTAGGGCCAGTCAAAGGA-3’ CAT: (F) 5’-TATGAGGAGCGGTTCGACTT-3’ (R) 5’-GCGTGTCGGAGTAGGAGAAG-3’

Real-time quantitative PCR amplifications were performed using the Light Cycler 480 (Roche). Three biological replications and at least 3 technical replication for each gene region were performed. The PCR was performed in a reaction mixture of 10 µL composed of 2–3 µL of cDNA, 0.4–0.8 µL of forward primer and reverse primer (10 pmol), 5 µL of LightCycler 480 SYBR Green I Master (Roche), and ddH2O. The standard curve was prepared from serial dilutions of control cDNAs. The following program was applied: initial polymerase activation: 95 °C for 10 min, then 45 cycles at 95 °C for 10 s, Tm °C for 10 s, and 72 °C for 5 s. The specificity of the PCR amplification was checked with a melt curve analysis (from 95 °C to Tm) following the final cycle of the PCR. PCR conditions were optimized for high amplification efficiency of >95% for all primer pairs used. The Ct (cycle threshold) values of the amplification curves were obtained between the 20th and 35th cycles. In melting curve analyses, overlapping single peak images were obtained and dimer presence was not detected.

Ct values were established by using the peak profiles. According to results obtained from Ct values, the relative expression levels were calculated by using REST 2009 software (http://www.gene-quantification.de/rest-2009. html) according to the 2–ΔΔCT algorithm. Reaction efficiency (RE) was considered as 1. Confidence interval (CI) was considered as 95%. The results of CAT and Cu/Zn SOD gene expression levels with the 2–ΔΔCT method (Livak and Schmittgen, 2001) were normalized using the expression value of the GAPDH house-keeping gene.

### 2.8. Statistics

Data obtained from analyses were evaluated statistically at the factorial level by means of variance analyses (ANOVA) and their significance levels (P ≤ 0.05 and P ≤ 0.01) were determined. All analyses and measurements were performed repetitively at least three times.

## 3. Results

Compared to the seedlings grown in the light as the control group, dark caused a considerable decrease in seedling length. Concentrations of 50 and 100 mg L–1 IAA sprayed onto leaves as a senescence inducer caused an increase in seedling length, while 200 mg L–1 AgNO3 did not cause a significant change in seedling length for seedlings grown in light and 200 mg L–1 AgNO3 treatment caused a significant increase in seedling length for seedlings with darkinduced senescence (P ≤ 0.05). AgNO3 treatment in the dark resulted in higher seedling length values compared to the control group. For seedlings treated with 50 and 100 mg L–1 IAA, AgNO3 treatment did not have a significant effect on seedling length (Table 1).

RWC values showed a limited change between 61% and 72% for all treatments. The control group plants grown in the light with no treatment gave the highest RWC values, whereas the lowest value was measured for plants treated with 50 mg L–1 IAA and 200 mg L–1 AgNO3 (Table 1). In terms of RWC values, the difference between plants treated with AgNO3 and plants not treated with AgNO was found 3 to be statistically insignificant. The difference between the control group and groups administered senescence inducers was not significant, as well.

Compared to controls, significant differences were found for seedlings treated with dark and IAA in terms of total chlorophyll values. Compared to the control plants grown in the light, dark and IAA treatments led to significant decreases in total chlorophyll amount (Table 1). The most significant decrease was found for the dark treatment. Chlorophyll loss increased as the concentration of IAA sprayed onto leaves increased, and 200 mg L–1 AgNO3 sprayed onto wheat leaves reduced chlorophyll loss for all groups. Chlorophyll amount in seedlings treated with AgNO3 together with dark showed 53% increase compared to seedlings treated with dark only. On the other hand, the highest chlorophyll amount was observed in seedlings treated with AgNO3 in light. In seedlings treated with 50 and 100 mg L–1 concentrations of IAA, AgNO3 treatment led to an increase in chlorophyll amount (Table 1).

Compared to the control plants grown in the light, β-carotene amount decreased with senescence inducers. The β-carotene loss was 27% with dark treatment, 21% with 50 mg L–1 IAA treatment, and 25% with 100 mg L–1 IAA treatment. Compared to the control group, IAA and dark treatments led to statistically significant differences in terms of β-carotene amount in seedlings (P ≤ 0.05). AgNO3 treatment led to an increase in terms of β-carotene amount in all groups, whereas the most significant increase was observed in seedlings treated with 50 mg L–1 IAA. However, in terms of β-carotene values, the difference between plants treated with AgNO3 and plants not treated with AgNO3 was found to be statistically insignificant (Table 1). Total xanthophyll value was found to be lower in dark- and IAA-treated groups compared to controls grown in the light. Total xanthophyll loss was about 76% in groups treated with 100 mg L–1 IAA. AgNO3 treatment increased total xanthophyll amounts in all groups. The rate of increase was higher in groups treated with dark and 100 mg L–1 IAA. The increase in total xanthophyll amount observed with AgNO3 treatment varied between 7% and 8% (Table 1).

Total antioxidant capacity significantly decreased in dark-induced senescence conditions. Total antioxidant capacity was found to be 43% lower in wheat plants left in the dark compared to the plants grown in light (Table 2). The effect of IAA treatment on antioxidant capacity was more limited. Compared to the control group, IAA and dark treatments led to statistically significant differences in terms of total antioxidant capacity in seedlings (P ≤ 0.05). AgNO3 led to a limited increase in total antioxidant capacity in controls and groups treated with IAA. Total antioxidant capacity increased in seedlings treated with AgNO3 in the dark compared to seedlings with no treatment and seedlings left in the dark (Table 2). Comparing two different concentrations of IAA, 50 and 100 mg L –1, total antioxidant capacity was found to be similar.

**Table 2:** 

Treatments	Antioxidant capacity (mg vit. C eq. FW)	Soluble phenolics (μg g–1 FW)	Soluble proteins (μg g–1 FW)	CAT activity (units g–1 FW)	Total SOD activity (units g–1 FW)	Cu/Zn- SOD (units g–1 FW)
Light	96.6 ± 5.1	3433 ± 76	4986.7 ± 61	108 ± 4.8	349 ± 10.1	148 ± 9.9
Light + AgNO3	127.5 ±10.6	3967 ± 37	5378.7 ± 98	138 ± 6.2	412 ± 15.4	209 ± 11.3
Dark	42.3 ± 2.1	1817 ± 76	3068.0 ± 72	60 ± 2.8	251 ± 15.2	81 ± 6.0
Dark + AgNO3	67.3 ± 5.1	2400 ± 32	3805.3 ± 25	92 ± 5.4	313 ± 25.3	135 ± 20.3
IAA(50)	71.9 ± 3.6	2200 ± 29	4133.3 ± 89	118 ± 9.4	389 ± 32.2	184 ± 31.3
IAA(50) + AgNO3	92.1 ± 4.3	2883 ± 31	4493.3 ± 56	138 ± 4.0	477 ± 7.6	237 ± 6.3
IAA(100)	59.3 ± 3.9	1973 ± 54	3640.0 ± 20	134 ± 5.6	421 ± 21.3	214 ± 13.3
IAA(100) + AgNO3	73.8 ± 3.6	2450 ± 80	4108.0 ± 63	148 ± 4.6	494 ± 33.0	269 ± 19.8
Statistics	Inducer (*)	Inducer (*)	Inducer (*)	Inducer (*)	Inducer (*)	Inducer (*)
	Silver (*)	Silver (*)	Silver (*)	Silver (*)	Silver (*)	Silver (*)

Soluble phenolics content decreased in wheat plants treated with dark and IAA compared to the control group. The decrease was 47% with dark treatment, 36% with 50 mg L–1 IAA treatment, and 43% with 100 mg L–1 IAA treatment. The decrease in soluble phenolics amount was not found to be significant in statistical tests (P ≤ 0.05). AgNO3 treatment was observed to significantly effect soluble phenolics amount. AgNO3 increased soluble phenolics levels significantly in all controls left in the light and the plants with dark- and IAA-induced senescence compared to plants not treated with AgNO3 (P ≤ 0.05). AgNO3 increased soluble phenolics levels by 15% for plants left in the light, 33% for plants left in the dark, 31% for plants treated with 50 mg L–1 IAA, and 24% for plants treated with 100 mg L–1 IAA (Table 2).

The most significant change in terms of total soluble protein amount in leaf tissues was observed in plants left in the dark. Soluble protein amount decreased by 38% in plants left in the dark compared to plants left in the light. IAA treatment led to a decrease in soluble protein level and the decrease was proportional to IAA concentration. It was found that 50 mg L–1 IAA treatment led to a decrease of 17%, whereas 100 mg L–1 IAA treatment led to a decrease of 28% (Table 2). AgNO3 treatment affected the soluble protein levels of the plants grown in the light; moreover, it mitigated soluble protein loss in plants left in the dark and treated with IAA. With AgNO3 treatment the improvement in soluble protein level was observed to be at its height in the leaves of plants left in the dark. AgNO 3 treatment increased soluble protein content by 24% in seedlings left in the dark (Table 2). AgNO3 led to a higher increase in terms of soluble protein amount in plants treated with IAA compared to plants not treated with IAA.

CAT activity values significantly decreased in plants left in the dark compared to controls and significantly increased in plants treated with 50 and 100 mg L–1 IAA. The differences between treatment groups in terms of CAT enzyme activity were found to be statistically significant (P ≤ 0.05). AgNO3 treatment increased CAT enzyme activity in plants left in the dark and plants treated with 50 and 100 mg L–1 IAA. Differences between controls, 50 and 100 mg L–1 IAA-treated seedlings, and seedlings left in the dark and seedlings treated with AgNO3 in terms of CAT enzyme activity were found to be statistically significant (P ≤ 0.05). AgNO3 led to an increase of 54% in terms of CAT activity in seedlings left in the dark. AgNO 3 led to an increase of about 16% in seedlings treated with 50 mg L–1 IAA, whereas it led to an increase of about 11% in seedlings treated with 100 mg L–1 IAA. That being said, the highest CAT activity was observed in seedlings treated with 100 mg L–1 IAA and AgNO3 (Table 2).

Total SOD activity values decreased in plants left in the dark compared to controls and increased in plants treated with 50 and 100 mg L–1 IAA. The differences between treatment groups in terms of SOD enzyme activity in leaves were found to be statistically significant (P ≤ 0.05). SOD activity of 349 units in controls reached 421 units with 100 mg L–1 IAA treatment (Table 2). Similar to total SOD values, Cu/Zn-SOD values decreased in the dark and increased with 50 and 100 mg L–1 IAA treatments (Table 2). AgNO3 led to an increase of about 25% in terms of SOD activity in seedlings left in the dark. In terms of SOD activity, AgNO3 led to an increase of about 22% in seedlings treated with 50 mg L–1 IAA, whereas it led to an increase of about 17% in seedlings treated with 100 mg L–1 IAA. That being said, the highest SOD activity was observed in seedlings treated with 100 mg L–1 IAA and AgNO3 (Table 2). AgNO3 treatment led to an increase in Cu/Zn-SOD activity in seedlings left in the dark and Cu/ Zn-SOD activity in seedlings treated with IAA.

**Figure 1:**
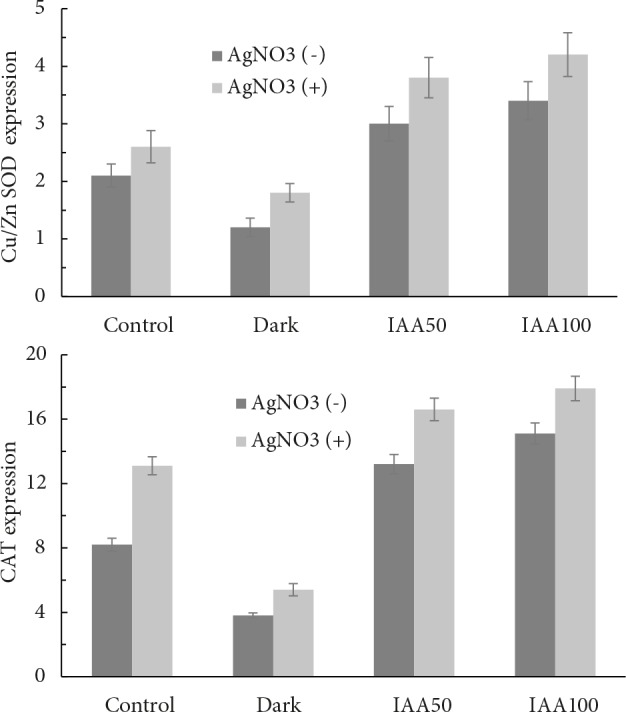
In wheat plants with dark-induced senescence and senescence induced with two different concentrations of IAA, 50 and 100 mg L–1, the effect of AgNO3 treatment at the concentration of 200 mg L–1 on relative expression levels of: a) Cu/Zn-SOD and b) CAT genes. Data represent the mean ± standard error of three independent experiments. Senescence inducers (dark, IAA), * P ≤ 0.05; silver (AgNO3 treatment), * P ≤ 0.05; inducer (dark, IAA) × silver (AgNO3 treatment), * P ≤ 0.05. GAPDH was used as the housekeeping gene.

Compared to controls in terms of Cu/Zn-SOD gene expression, AgNO3-treated groups showed a significant increase (P ≤ 0.05). Dark treatment led to a significant decrease in Cu/Zn-SOD gene expression compared to controls (Figure 1a). IAA treatment at concentrations of 50 mg L–1 and 100 mg L–1 increased Cu/Zn-SOD gene expression in a statistically significant manner (P ≤ 0.05) (Figure 1a). The highest Cu/Zn-SOD gene expression was observed in plants treated with both 100 mg L–1 IAA and AgNO3 (Figure 1a). Compared to controls, CAT gene expression increased significantly with 50 mg L –1 and 100 mg L–1 IAA treatment (P ≤ 0.05), whereas dark led to a significant decrease in CAT gene expression (P ≤ 0.05) (Figure 1b). AgNO3 treatment led to a significant increase in CAT gene expression in controls, plants left in the dark, and plants treated with IAA (P ≤ 0.05) (Figure 1b).

## 4. Discussion

Leaf senescence is a crucial and obscure process in the life period of plants, throughout which cells undergo major changes at structural and functional levels (Lambert et al., 2017) .

2,4-D-induced leaf senescence in beans was inhibited with supplementation of Ag+ ions in the form of AgNO3 or AgNPs. 2,4-D mitigated growth and stimulated senescence at high concentrations. 2,4-D treatment together with 100 µM AgNO3 produced a seedling length close to that of the control (Karuppanapandian et al., 2011) . Treatment with natural and synthetic auxins in increasing proportions caused a decrease in root and trunk length in mustard and pea (Hansen and Grossmann, 2000; Swarup et al., 2007) . In this study, the decrease in seedling length observed when the seedlings were left in the dark was inhibited with AgNO3 treatment (Table 1).

In a study conducted with two different maize varieties with 80-day and 100-day lifespans, RWC decreased in both varieties after tasseling; however, the variety with 100-day lifespan had higher RWC (Prochazkova et al., 2001) . This may be associated with later onset of senescence in the variety with a lifespan of 100 days compared to the variety with 80 days of lifespan since RWC decreases as senescence progresses. In this study, RWC was not significantly affected by dark, IAA, or AgNO 3 treatments (Table 1), which indicates that RWC is not an important parameter in the dark- and IAA-induced senescence process.

The decrease in total chlorophyll content was higher in the early aging line compared to a normal aging line in the leaf senescence process in wheat (Hongwei et al., 2014). In French bean leaves during natural and dark-induced senescence, chlorophyll content decreased (Lambert et al., 2017) . In beans, chlorophyll content decreased by approximately 50% in seedlings treated with 500 µM 2,4D compared to controls (Karuppanapandian et al., 2011) . Treatment with natural and synthetic auxins in increasing proportions caused chlorophyll loss in mustard and pea (Hansen and Grossmann, 2000; Swarup et al., 2007) , while 50 and 75 mg L–1 IAA sprayed onto beans led to an increase in terms of chlorophyll content compared to controls (Sadak et al., 2013) . Furthermore, 0.01 and 10 µM IAA solutions sprayed onto sunflower cotyledons were found to trigger chlorophyll destruction ( Gören and Çağ, 2007 ). IAA treatment at high concentrations was found to accelerate chlorophyll loss in leaf disks of lettuce (Aharoni, 1989) . However, in another study conducted with wheat, IAA treatment was reported to prevent chlorophyll loss in chloroplasts both in vivo and in vitro (Misra and Biswall, 1980) . It was found that chlorophyll loss in wheat leaves was associated with IAA concentration. Chlorophyll loss in the dark- and IAA-induced senescence process was reduced with AgNO3 treatment, which shows that chlorophyll destruction triggered by senescence is prevented in the presence of Ag+ ions.

IAA sprayed onto beans caused an increase in the amount of carotenoids (Sadak et al., 2013) . In darkinduced senescence, treatment of Tropaeolum majus leaves with IAA accelerated carotenoid loss ( Karataş et al., 2010 ). In this study, it was observed that IAA treatment led to β-carotene loss (Table 1). Carotenoid content was significantly reduced in leaves left in the dark compared to controls. This loss was 27% in plants left in the dark compared to controls (Table 1). Leaves of Pelargonium zonale left in the dark and treated with AgNPs showed an increase in carotenoid amount. However, AgNPs caused a decrease in carotenoid amount at 80 mg L–1 and higher concentrations (Hatami and Ghorbanpour, 2014). This decrease may be due to the toxic effect of AgNPs at 80 mg L–1 and higher concentrations. In this study, AgNO3 treatment led to an increase in terms of β-carotene amount in all groups (Table 1).

Total antioxidant capacity is used to refer to all antioxidants for removing free radicals in plant samples (Mitrovic and Bogdanovic, 2009) . Total antioxidant capacity significantly decreased in dark-induced senescence conditions. AgNO3 led to an increase in total antioxidant capacity in controls and groups treated with IAA. In seedlings left in the dark and treated with AgNO 3, antioxidant capacity showed a significant increase of 59% compared to seedlings left in dark (Table 2).

Increasing concentrations of silver nanoparticles caused a decrease in phenol and tannin content in plant cells due to the toxic effect of silver (Fatemeh et al., 2014). In this study, AgNO3 treatment was observed to significantly effect soluble phenolics amount. AgNO 3 treatment increased soluble phenolics levels significantly in controls left in the light and the plants with dark- and IAA-induced senescence compared to plants not treated with AgNO . AgNO3 increased soluble phenolics levels 3 by 15% for plants left in the light, 33% for plants left in the dark, 31% for plants treated with 50 mg L–1 IAA, and 24% for plants treated with 100 mg L–1 IAA (Table 2). IAA sprayed onto beans caused an increase in the amount of total phenolic compounds (Sadak et al., 2013) . In this study, soluble phenolics content decreased in plants treated with dark and IAA compared to the control group.

Leaf senescence is characterized with decreased total protein content. While many proteins degrade during senescence, some remain intact. The decrease in protein amount was more rapid in early aging lines compared to normal aging lines in wheat (Hongwei et al., 2014). Protein content decreased in Tropaeolum majus leaves kept in the dark and treated with IAA during senescence ( Karataş et al., 2010 ). In French bean leaves during natural and darkinduced senescence, protein content decreased (Lambert et al., 2017) . Auxins mitigate protein loss in the senescence process by stimulating protease inhibitors and increase the expression of protease inhibitor genes in the senescence process ( Karataş et al., 2010 ). In this study, total soluble protein amount significantly decreased in leaf tissues of seedlings left in the dark compared to controls (Table 2). IAA treatment led to a decrease in soluble protein level and the decrease was proportional with IAA concentration. IAA causes a decrease in soluble protein amounts at high concentrations, whereas IAA administered in normal concentrations may decrease protein loss in the senescence process.

ROS concentration increases together with the decrease in SOD and CAT activities; thus, lipid peroxidation, senescence, and cell death increase (Chakrabarty et al., 2007) . According to studies conducted with different plant species, CAT enzyme activity continually decreased from the onset of senescence in cucumber and bean cotyledons (Manoharan et al., 2005) . The activity of CAT enzyme increased until 25 days after tasseling in maize; however, it decreased subsequently (Prochazkova et al., 2001) . CAT activity increased at the beginning of senescence; however, a decrease was observed in CAT enzyme activity as senescence progressed. In wheat, CAT enzyme activity was found to be lower in early aging lines compared to normal aging lines in samplings performed with different time intervals (Hongwei et al., 2014). Meanwhile, IAA in high concentrations may lead to a decrease in CAT enzyme activity by inducing senescence. In this study IAA and AgNO3 treatment led to an increase in CAT enzyme activity by slowing down the senescence process. However, in dark-induced seedlings, CAT enzyme activity decreased (Table 2).

Although total SOD activity increased at the beginning of senescence in leaves of tobacco (Dertinger et al., 2003) and maize (Prochazkova et al., 2001) , it decreased as senescence progressed. SOD enzyme activity showed a continuous decrease with the onset of senescence in wheat leaves (Srivalli and Khanna-Chopra, 2001) . Cu/Zn-SOD and Mn-SOD activity increased at the beginning of senescence in bean cotyledons (Prochazkova and Wilhelmova, 2007) , but decreased as senescence progressed. Mn-SOD activity continually decreased in pea leaves (Jimenéz et al., 1998) from the onset of senescence. 2,4-D sprayed onto leaves of pea led to an increase in superoxide and hydrogen peroxide radicals and formation of oxidative stress by causing protein destruction, and SOD enzyme activity increased significantly in spite of this (Romero-Puertas et al., 2004) . In wheat, SOD and CAT enzyme activities were found to be lower in early aging lines compared to normal aging lines in samplings performed with different time intervals (Hongwei et al., 2014). In this study, IAA and AgNO3 treatment lead to an increase in SOD and Cu/Zn- SOD enzyme activity by slowing down the senescence process. However, in dark induced seedlings, SOD and Cu/Zn-SOD enzyme activity decreased (Table 2).

After 20 h of incubation in the dark, expression of mitochondrial Mn-SOD, chloroplastic Fe-SOD, and cytoplasmic Cu/Zn-SOD enzyme transcripts decreased in both young and aging barley leaves (Casano et al., 1994) . Dark led to a decrease in Cu/Zn-SOD gene expression compared to controls (Figure 1a). In pea, 2,4-D treatment led to an increase in Mn-SOD transcript level and a decrease in Cu/Zn-SOD transcript level (Romero-Puertas et al., 2004) . In this study, IAA and AgNO3 treatments led to a significant increase in Cu/Zn-SOD gene expression compared to controls (Figure 1a). An increase might have occurred in antioxidant capacity since IAA and AgNO3 may have senescence-inhibiting effects in plants at certain concentrations and the increase in Cu/Zn-SOD gene expression may be a result of this mechanism. In wheat, Cu/Zn-SOD gene expression was at the same level in early and late aging lines right after expansion of the flag leaf. However, it was lower in early aging lines compared to normal aging lines after expansion of the flag leaf. Lower Cu/Zn-SOD gene expression in early aging lines compared to normal aging lines may be due to the fact that senescence signs emerge earlier in early aging lines compared to normal aging lines (Hongwei et al., 2014). Compared to seedlings left in the dark, Cu/Zn-SOD gene expression increased in seedlings left in the dark and treated with AgNO3 as well (Figure 1a). This increase in Cu/Zn-SOD gene expression may be associated with the AgNO3 treatment mitigating the effect of ethylene and delaying senescence (Figure 1a).

The decrease in CAT gene expression was more rapid in early aging lines compared to normal aging lines in wheat with the onset of senescence (Hongwei et al., 2014). The CAT gene family consists of three genes called CAT1, CAT2, and CAT3 in the Arabidopsis genome (Frugoli et al., 1996) . While CAT2 gene expression decreases in the senescence process, CAT3 gene expression is stimulated by senescence (Zimmermann et al., 2006; Xin g et al., 2007 ). The expression of the CAT3 gene is induced with dark and increases in leaves with senescence (Du et al., 2008) . The CAT3 gene may be involved in regulation of oxidative homeostasis throughout senescence (Park et al., 1998; Zimmermann et al., 2006) . In this study, CAT gene expression significantly decreased in plants left in the dark compared to controls. In pea, 2,4-D treatment led to an increase in CAT transcript level (Romero-Puertas et al., 2004) . In this study, IAA treatment led to an increase in CAT gene expression. Although IAA has senescenceinhibiting effects up to certain concentrations, it may have senescence-inducing effects in increased concentrations. Also, the increase or decrease in CAT gene expression may vary depending on IAA concentration. AgNO3 treatment led to an increase in CAT gene expression in all groups (Figure 1b).

CAT activity increased at the beginning of senescence; however, a decrease was observed in CAT enzyme activity as senescence progressed (Hongwei et al., 2014). Cu/Zn-SOD and Mn-SOD activity increased at the beginning of senescence in bean cotyledons (Prochazkova and Wilhelmova, 2007) , but decreased as senescence progressed. In this study, which applied IAA at 50 and 100 mg L–1 as senescence-inducing stimulants, compared to the control, the enzyme activity of CAT, SOD, Cu/ZnSOD and CAT, and Cu/Zn-SOD gene expression increased (Table 2; Figures 1a and 1b). However, IAA treatment decreased the parameters associated with the progression of the senescence process, such as total chlorophyll, β-carotene, total xanthophyll, total antioxidant capacity, soluble phenolics, and soluble proteins when compared with control seedlings (Table 1).

Findings obtained from this study showed that the senescence process was related to changes in the level of antioxidant compounds and enzymes. Dark induction causes a decrease in all antioxidant parameters, while with IAA application some antioxidant parameters increased (enzyme activity of CAT, SOD, Cu/Zn-SOD and CAT, Cu/ Zn-SOD gene expression) while some (total chlorophyll, β-carotene, total xanthophyll, total antioxidant capacity, soluble phenolics, and soluble proteins) decreased in the senescence process. AgNO3 treatment showed a senescence-delaying effect. However, conducting a similar study with different concentrations of IAA and AgNO 3 and performing time-dependent analyses with harvest may produce more detailed findings about effects of IAA and AgNO3 ions on the senescence process.

## Acknowledgment

This study was supported by the Scientific Research Projects Unit of Mersin University (Project No. BAP 2015TP2-1045).
